# Developing Suicide Prevention Tools in the Context of Digital Peer Support: Qualitative Analysis of a Workshop With Multidisciplinary Stakeholders

**DOI:** 10.2196/47178

**Published:** 2023-09-20

**Authors:** Bethany Cliffe, Jessica Gore-Rodney, Myles-Jay Linton, Lucy Biddle

**Affiliations:** 1 Population Health Sciences Bristol Medical School University of Bristol Bristol United Kingdom; 2 Tellmi London United Kingdom

**Keywords:** digital interventions, smartphone app, suicide prevention, mental health, mobile phone

## Abstract

**Background:**

Suicide is the fourth leading cause of death among young people aged 15-29 years worldwide and suicide rates are increasing. Suicide prevention strategies can be effective but young people face barriers to accessing them. Providing support digitally can facilitate access, but this can also pose risks if there is inappropriate or harmful content. Collaborative approaches are key for developing digital suicide prevention tools to ensure support is appropriate and helpful for young people. Tellmi (previously MeeToo) is a premoderated UK-based peer-support app where people aged 11-25 years can anonymously discuss issues ranging from worries to life challenges. It has procedures to support high-risk users, nevertheless, Tellmi is interested in improving the support they provide to users with more acute mental health needs, such as young people struggling with suicide and self-harm ideation. Further research into the best ways of providing such support for this population is necessary.

**Objective:**

The aim of this study is to explore the key considerations for developing and delivering digital suicide prevention tools for young people aged 18-25 years from a multidisciplinary perspective, including the views of young people, practitioners, and academics.

**Methods:**

A full-day, in-person workshop was conducted with mental health academics (n=3) and mental health practitioners (n=2) with expertise in suicide prevention, young people with lived experience of suicidal ideation (n=4), and a computer scientist (n=1) and technical staff from the Tellmi app (n=6). Tellmi technical staff presented 14 possible evidence-based adaptations for the app as a basis for the discussions. A range of methods were used to evaluate them, including questionnaires to rate the ideas, annotating printouts of the ideas with post-it notes, and group discussions. A reflexive thematic analysis was performed on the qualitative data to explore key considerations for designing digital suicide prevention tools in the context of peer support.

**Results:**

Participants discussed the needs of both those receiving and providing support, noting several key considerations for developing and delivering digital support for high-risk young people. In total, four themes were developed: (1) the aims of the app must be clear and consistent, (2) there are unique considerations for supporting high-risk users: (subtheme) customization helps tailor support to high-risk users, (3) “progress” is a broad and multifaceted concept, and (4) considering the roles of those providing support: (subtheme) expertise required to support app users and (subtheme) mitigating the impact of the role on supporters.

**Conclusions:**

This study outlined suggestions that may be beneficial for developing digital suicide prevention tools for young people. Suggestions included apps being customizable, transparent, accessible, visually appealing, and working with users to develop content and language. Future research should further explore this with a diverse group of young people and clinicians.

## Introduction

Suicide is the fourth leading cause of death among those aged 15-29 years [1], and data show that 1010 people aged 30 years or younger died by suicide in England in 2021 [2]. A rise in suicide rates has also been witnessed in recent years, with a 7.9% increase in adolescent suicides per year reported between 2010 and 2017 in England and Wales [3]. This highlights the urgency surrounding the need for effective suicide prevention strategies.

A systematic review and meta-analysis of 16 controlled studies found that suicide prevention strategies can be effective at reducing both suicide attempts and deaths by suicide [4]. In particular, there is evidence to suggest that lethal means restriction, psychotherapy, and school-based awareness campaigns can be effective. However, the evidence suggests that no suicide prevention strategy seems more effective than others [5]. Importantly, survivors of a suicide attempt have reported not seeking support due to a lack of available resources, a distrust of mental health professionals, and not having enough time [6]. This suggests that, while suicide prevention strategies may be effective, they can be difficult to access. This issue is particularly prevalent among young people; a case series examining suicide amongst those aged 20 years or younger in England found that around half did not have contact with mental health or social services prior to their death [7]. This is troubling and underscores the need to ensure young people at risk of suicide can access support that is not subject to barriers as described above.

Digital tools such as smartphone apps are one option for facilitating suicide prevention among young people [8] given that around 96% of young people aged 16-24 years in the United Kingdom own smartphones [9]. Evidence is emerging from systematic reviews that digital tools, such as self-guided smartphone apps or web-based therapy, can be immediately effective in reducing suicidal ideation, particularly those that directly target suicide, and that the effects are comparable to face-to-face interventions [10]. Moreover, digital tools are not typically subject to the same barriers as professional support, such as stigma or waiting lists, and they are accessible 24/7. However, there are also risks to digital tools; young people using web-based communities to receive support during crises reported being triggered by negative interactions with peers [11]. Similarly, a systematic review identified that some suicide prevention apps included dangerous advice encouraging suicide or other harmful behaviors [12]. This emphasizes a need to ensure that digital support is provided in a way that is safe and does no harm.

Taking a collaborative approach to designing and developing digital suicide prevention tools is key to ensuring that they are safe and effective [13]. Individuals with lived experience have expertise in how those with similar needs can be best supported, and coproduction can therefore help to develop more successful, engaging, and acceptable mental health support while also empowering the individual [14]. Other key stakeholders such as industry partners and academics and clinicians with knowledge of suicide prevention can also contribute valuable insight to help ensure apps are evidence-based and appropriate [15]. This is important considering a review of app stores found that only 3% of available mental health apps had an evidence base [16]. The expertise of a range of stakeholders therefore should be sought when developing suicide prevention tools.

Tellmi (previously known as MeeToo) is a free-to-use premoderated UK-based peer-support app where young people aged 11-25 years can talk to others of a similar age anonymously about issues ranging from worries to life challenges. At Tellmi, a peer is an individual who downloads, signs up, and uses the Tellmi app organically. They are considered a peer due to being of a similar age to the users they engage within the app and their lived experiences, which is why they are highly valuable as peer supporters. Peer support is theorized to be beneficial for young people because the support is based on a mutual understanding of lived experiences that enables them to give and receive help rooted in a common understanding of psychological and emotional pain [17]. It is therefore argued that people with lived experiences of suicide attempts can provide vital support to those contemplating suicide and battling their suicidal thoughts [18].

In Tellmi internally trained moderators risk assess every post and reply before they are published and seen in the feed by other age-banded users. A user’s post or reply will be escalated to the in-house counselors for a single-session intervention if the trained moderators believe that the content of a user’s post or reply suggests that their life is at risk. In-house counselors can request support from emergency services if they deem that the user’s life is at imminent risk.

An evaluation of the app has identified that Tellmi helps users find new ways of managing their difficulties and that peer support within the app helps users feel less alone and more connected with those experiencing similar issues [19]. However, Tellmi’s support for young people experiencing severe distress, including suicidal thoughts, is less developed. Given the finding that digital tools for suicide prevention can be equally as effective as face-to-face interventions [10], Tellmi aims to extend its offering to better cater to this population.

To facilitate this, a collaborative workshop was held to seek the input of key stakeholders in guiding this process by discussing possible adaptations to the app that could aid in suicide prevention.

Principles from these discussions were identified that can be applied broadly to the development of digital suicide prevention tools. Consequently, this paper presents key considerations and suggestions for developing and delivering suicide prevention tools in the context of digital peer support for young people aged 18-25 years.

## Methods

### Study Design

An in-person workshop lasting 6.5 hours was run at a medium-sized research-intensive university in England in September 2022. This was part of a broader industry and academic collaborative project aiming to (1) develop the app so that it can aid in suicide prevention and (2) evaluate its use with those who may be at risk of suicidal thoughts or behaviors. The aim of the workshop was to evaluate candidate suicide prevention ideas for inclusion in the app, and this paper focuses on the findings that can be broadly applied to those developing similar tools.

### Recruitment

In total, 16 stakeholders attended the workshop including mental health academics (n=3) and mental health practitioners (n=2) with expertise in suicide prevention, young adults with lived experience of suicidal ideation and providing peer support (n=4), a computer scientist (n=1), and Tellmi technical staff (n=6). The academics were the research team who also facilitated the workshop. To recruit participants, the research team emailed contacts in existing academic and research networks asking them to share study information, using a snowball approach. Adverts were also placed on Twitter to reach a wider audience. Those interested were asked to contact the research team via email, who sent participants an information sheet and a sign-up form for them to register their intent to attend the workshop. The young people with lived experience received £150 (US $188.37) worth of vouchers each as compensation for their time. No other participants received payment but were provided with a meal at lunchtime and refreshments throughout the day. The rationale for renumerating the young people was to ensure that discussions were heavily informed by lived experience, whereas for staff, this may more naturally fit within their work.

### Ethical Considerations

This study received ethical approval from the University of Bristol Faculty of Health Sciences Ethics Committee (12159). Participants were provided with information sheets in advance of the workshop and were asked to sign a consent form on the day. Participants with lived experience were provided with a list of well-being resources and services. They were also offered support from one of Tellmi’s trained counselors following the workshop if they required extra support.

Throughout this paper and during workshop discussions, the term “high-risk user” refers to a Tellmi app user experiencing severe distress, including possible suicidal thoughts or intent.

### Workshop Structure

The workshop was focused on exploring consensus among stakeholder groups. Expert workshops [20,21] primarily create opportunities for focused discussion that draws on lived experience and learned expertise. The workshops used independent post-it note writing, small group discussions, and larger group discussions to explore consensus.

**Figure 1:**
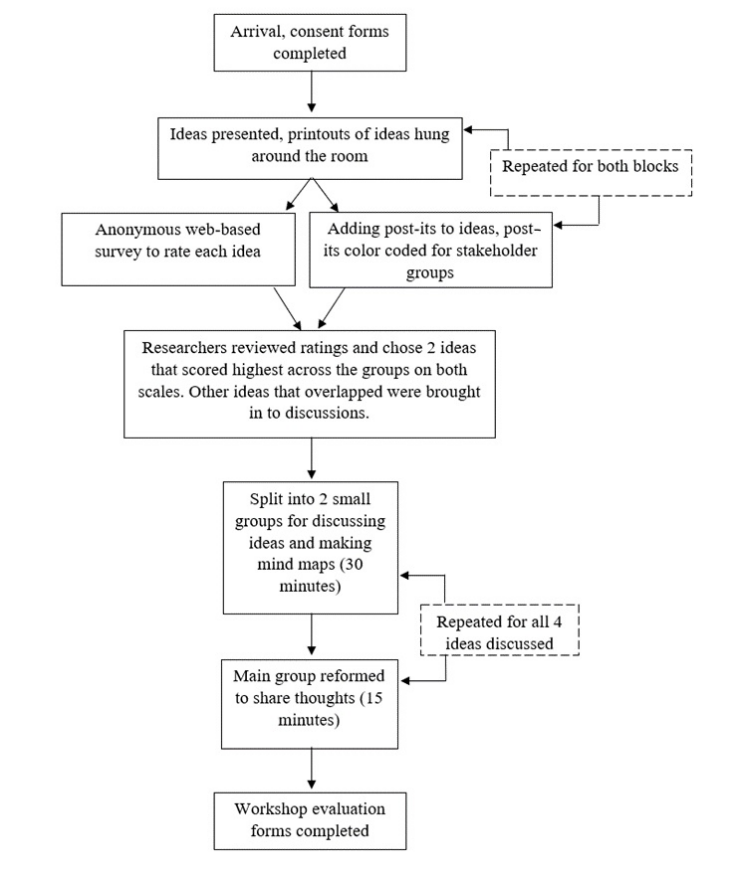
Flow diagram showing the procedure of the full-day workshop.

The workshop opened with a brief introduction during which participants were informed about the structure (Figure 1) and purpose of the day and were reminded to draw on their experiences and expertise when evaluating the ideas.

Tellmi technical staff presented 14 candidate changes or additions to the app (Multimedia Appendix 1) that may have the potential to support high-risk users. The candidate ideas were initially devised from the findings of a literature review that aimed to identify and understand what features and content young people experiencing suicidal ideation desire from web-based suicide interventions. The ideas identified from the literature were then scored against a 5-point (1 difficult and 5 easy) selection criterion, which considered the scope, time to develop, cost to deliver, impact, evidence, risk, and scalability of each idea by Tellmi technical staff, resulting in 14 candidate ideas for discussion at the workshop. A sample of these ideas is presented in Multimedia Appendix 1; however, as some of these ideas are yet to be implemented, they are unable to be detailed here. The ideas were presented in 2 separate blocks: block 1 focused on encouraging engagement with the app among high-risk users, and block 2 focused on ensuring the safety of high-risk users. Using a Survey Monkey questionnaire, participants were then asked to rate each idea on 2 scales. Scale 1 evaluated the potential positive impact from 1 (no positive impact) to 4 (very positive impact). Scale 2 evaluated the extent to which the ideas needed to be discussed before implementation, from 1 (no discussion required) to 4 (needs much discussion). These informed which ideas were discussed in detail in the afternoon.

Workshop evaluation forms were completed at the end. Please see Multimedia Appendix 2 for further information.

### Data Collection

Quantitative data included the idea ratings and workshop evaluation, qualitative data included free text comments left on post-it notes on A3 paper printouts of the slides presenting each idea. Further qualitative data were collected from A1 paper mind maps created during small group discussions that had the idea in the middle and the groups’ free-text thoughts written around it, discussion transcripts, and written feedback on the workshop evaluation form. This paper reports on the broader messages about app-based suicide prevention for young people arising from the workshop rather than on specific ideas, therefore the individual ratings are not reported here but can be found in Multimedia Appendix 1.

### Data Analysis

Post-it notes, mind maps, and discussion transcripts were analyzed using reflexive thematic analysis following the 6 steps outlined by Braun and Clarke [22,23]. The data were all read through for familiarization and discussed within the broader research team to identify common ideas. Two authors (BC and JGR) coded one transcript initially using the comments function of Microsoft Word. Any disagreements in coding between researchers were raised within group discussions exploring alternative interpretations of the data. They then coded the remaining data and discussed possible themes. Codes were compiled in a Microsoft Excel sheet and grouped into themes. This was an iterative process informed by group discussions, and mind maps were also made to visually explore possible themes. The data was then checked again to ensure that it was adequately captured by the themes. Finally, definitions for the themes were written and again discussed with the broader research team. Please see Multimedia Appendix 3 for examples of codes.

## Results

In total, 4 themes were developed that encompass recommendations for developing suicide prevention tools in the context of digital peer support.

### Theme 1: The Aims of the App Must Be Clear and Consistent

Participants emphasized that the aims of the app should be clear so that app users know exactly what support they can expect to receive. Additionally, the aims of the app and any new features should remain true to their intended purpose. In this instance, participants discussed how an app based on peer support should not try to provide services that diverge from what is advertised, such as counseling, as this would become “not what it says on the tin.” First, this may contradict what users want from the app—if they have signed up to receive peer support, they may not want something more aligned with counseling and to be approached in this way could be frightening or embarrassing:

It must be quite scary for the young person. Like, their heart jumping. "Oh, a counsellor’s coming to talk to you".

Young person

Second, it could leave app users confused about what the app offers and can sustain long-term. This may undermine their trust in it and could also negatively impact their well-being:

You guys [Tellmi technical staff] were saying that you’re not a counselling service and you didn’t want to give false expectations which I think is really important to uphold because a lot of people, especially with young people...they don’t need another person coming in and perhaps, unintentionally, setting those expectations that actually you guys aren’t set up to meet... the last thing this service wants to do is let them down.

Young person

Consequently, the aims of the app should be clearly communicated to users. This includes clarity around who is providing support, whether it be a peer or someone who may be qualified or trained in providing well-being support. Moreover, participants discussed how the app’s aims should be consistently transparent with users throughout use. For example, if an app user is waiting for a post to be moderated, it should be clearly communicated to them why they are waiting, and an estimate provided for how long they may be waiting for.

I think what’s been fairly evident so far is that being clear and honest about all the things that are going on in the app, as much as can be, is of high importance.

Computer scientist

### Theme 2: There Are Unique Considerations for Supporting High-Risk Users

#### Theme Overview

The workshop emphasized that supporting the target population of high-risk users must be carefully considered due to the unique challenges they may face. For example, participants discussed how difficult it can be to engage with complex or demanding content during a crisis, so any suggested activities within the app need to be accessible:

I think with some points of crisis where we know that, sort of, you shut down, your brain’s almost like, you can’t make decisions, you know, you’re not really sure what to do. Even if you could make the decision to have a bath, you can’t move, you’re like running a bath is too much at the moment.

Participant 1, Young person

You could provide really simplistic activities.

Participant 2, Young person

Quite passive things so that it’s more listening to something like riding the waves, sort of audio.

Participant 1, Young person

Support must therefore be designed to be accessible to high-risk users. It was first suggested that working directly with high-risk users to develop content can ensure that it is appropriate. This included collaborating to develop sensitive and appropriate language to avoid stigmatizing or triggering users. Second, any content that requires sustained user engagement should be simplistic, especially for those who may be struggling with motivation:

Especially, depression and things and you’re really struggling to kind of see a point to life and you don’t really want to be filling out these surveys like you know, because that’s quite like, it requires a bit of motivation to be honest to do that and then, on your worst day, arguably, you’re not going to want to do that.

Young person

It was also discussed how visually appealing content may help to engage high-risk users with tools designed to keep them safe during a crisis. Similarly, departing from content that is overly clinical was recommended, for example, avoiding words like “crisis” or “high-risk” within the app.

With regards to what you were saying about the NHS care plan, because it’s a peer group, do you think it’ll be useful to not make it exactly like a professional care plan?

Participant 1, Tellmi technical staff

I’m imagining something more fun to look at and something you want to look at because care plans are so clinical and often aren’t actually that helpful...I have a care plan and when I was feeling well one day, I drew it up into an illustration and it’s the only thing I look at. I’ve never looked at the official care plan. Why would I? It’s boring. It’s dull. It’s the worst thing.

Participant 2, Young person

Another example is that high-risk users need to feel listened to and cared for. Hiding their high-risk posts can make them feel segregated from the rest of the mental health community and punished for sharing their feelings. It was discussed how having a specialist, trained peer group to provide support to higher-risk posts could be valuable, but this would need to be clearly communicated to users, as discussed above. The well-being of those providing support must also be considered, which is discussed in theme 4.

#### Subtheme: Customization Helps Tailor Support to High-Risk Users

Given their unique needs, providing high-risk users with functions that allow them to customize the app was seen as important for ensuring that they remain in control of the support they receive, while also ensuring that the content shown is appropriate and relevant.

Something that came across was personalization, which seemed to be something that would be really nice to have, and being able to, essentially, plan for when you’re in a crisis would probably maybe make you feel slightly more supported by yourself on the app, and you’ve then got things in front of you that are exactly the things that you actually want to be looking at.

Tellmi technical staff

Participants discussed the benefits of enabling users to create personalized care plans, toolboxes with distractions or well-being activities, and bookmark preferred helplines to contact. Further to the types of content, being able to personalize how the content is visually presented was also favored as individuals may find different formats more accessible. This again ensures that they remain in control of the content they access.

Sometimes reading’s hard when you’re in a crisis. You need to have an option for just images.

Young person

Throughout discussions, participants were clear that “high-risk users” are not a homogeneous group and it should not be assumed that their needs are the same. Being able to customize and personalize the app also acknowledges this heterogeneity, allowing support to cater to people with different needs or circumstances. Moreover, peoples’ experiences of distress vary across time, meaning they may want different support at different times:

People do go through different states, don’t they? We go through phases where we think we want a bit more support or less support.

Mental health practitioner

### Theme 3: “Progress” Is a Broad and Multifaceted Concept

Discomfort around the concept of “progress” underpinned discussions around apps being used to understand or chart improvements in well-being. This inspired conversations about how progress could be more appropriately conceptualized. Tellmi is an app that embodies and encourages peer support as a mechanism for improving aspects of mental health, confidence levels, and young people’s abilities to seek support for their own mental well-being experiences. Though Tellmi does not have any features to measure a user’s mental well-being progression, the ideas of implementing ways of measuring progress into the app have been discussed. First, participants emphasized that progress is not linear:

We’ve sort of found this in like lived experience accounts, is reflecting the fact it’s not some linear thing and that it’s got ups and downs and, you know, that that’s actually normal.

academic

Thinking about progress linearly can be discouraging for people who are experiencing setbacks. This may be particularly relevant when progress is charted visually:

I don’t know about you, but I personally wouldn’t want to see like a graph or a chart or a visual representation of progress because...

Participant 1

It might be demoralizing.

Participant 2

Yeah.

Participant 1, Young person

Progress was understood as multifaceted, meaning different things to different people. For the participants with lived experience, it encompassed learning skills, having time for personal reflections, feelings of gratitude, feeling supported, sleeping well, being social, and being active, for example;

I think something worth exploring is what if we were to consider learning as a progress... So I may feel like really bad but at the same time I’ve learned something good.

Young person

It was noted that formal progress tracking within apps could deter user engagement, especially if linear progress is not being made.

They’re not going to carry on engaging if they’re just you know, they’re seeing the progress, there’s no progress, what does that then do?

Mental health practitioner

### Theme 4: Considering the Role of Those Providing Support

#### Theme Overview

While the needs of app users were focal in workshop discussions, the role of those providing support (eg, volunteers, specialist peers, or counselors) was also considered. This included the experience and skills required of them to be able to help users, as well as how they themselves could be supported in this challenging role.

#### Subtheme: Expertise Required to Support App Users

During the workshop, it was discussed whether high-risk users would need more specialized in-app support provided by staff or volunteers who are trained for the role. Participants raised concerns about assuming that individuals would be well equipped with the skills needed to provide support based solely on their qualifications. A participant reflected on how even having 2 master’s degrees was not enough for them to be able to handle supporting someone with their mental health.

If you were telling me, when I was doing the master’s, to do this role [providing peer support to high-risk young people], I would be all excited, but when I’m actually faced with that kind of conversation, I would not be very effective. Potentially, I would get really anxious as well.

Mental health practitioner

Consequently, it was noted that those providing specialized support to high-risk users should have lived experience. However, participants highlighted the risks of this as the individual providing support may find this triggering or be reminded of difficult personal experiences.

So I think as someone with lived experience, if I was in that role and something came up that I couldn’t... relate to is the wrong word, but that was, there was a parallel with an experience I’ve had, I might find that brings up a lot of memories and I’d find that really, really difficult.

Young person

#### Subtheme: Mitigating the Impact of the Role on Supporters

Discussions also raised the need for individuals in supportive roles to receive adequate help to reduce the impact that this role could have on their own mental well-being. This was particularly true for those who may have lived experiences of their own.

Consequently, one-to-one check-ins and signposting to mental health services were deemed necessary.

So I think you would need to have some kind of separate one-to-one chat with them [the individual providing support], or another service that they can be signposted to.

Young person

Participants also highlighted that it should be made very clear what would be expected of those providing specialist support so that they would be able to assess whether their capabilities and preparedness were adequate for the role.

You would need to almost have a one on one conversation and really kind of give them a full picture of what they’re dealing with... And if they’re signing up for this they should be aware that it isn’t easy and it may be triggering, and if they don’t feel like they are ready for that then maybe wait until they take on that responsibility.

Young person

Finally, helping those providing support to feel valued was emphasized, for example, through payment, providing employment references, or accreditation (for volunteers).

## Discussion

### Principal Results and Implications

This workshop identified key considerations and suggestions for developing and delivering suicide prevention tools to high-risk young people in the context of digital peer support. Notably, people in distress face unique challenges that must be addressed so that apps are appropriate and users can effectively engage with them. App customization and working with high-risk users to develop content and language can facilitate this. Communicating clearly and transparently with app users is also essential so that their expectations about the support available are managed. Participants argued that improvements in well-being are not adequately captured by the concept of “progress.” A broader understanding that acknowledges skills, lifestyle changes, and feelings is warranted. Finally, the well-being of those providing support must also be protected; ensuring they feel valued in their role, knowing what is expected of them, and feeling able to provide it can contribute to this. Tellmi will use this feedback in the decision-making of developing and improving app features.

### Comparison With Prior Work

Using co-design workshops with young people to inform the development of peer support for suicide prevention, Libon et al [8] identified the benefits of including youths in designing and implementing peer support. Throughout discussions in this study, participants suggested working with app users to develop content that is appropriate and appealing. Co-design of interventions is increasingly recognized as key for producing appropriate support [24], and that was reiterated here. The finding that there are unique considerations for supporting high-risk users adds further credence to this.

App customization was suggested for providing appropriate support. The benefits of app customization options have also been acknowledged by young people in co-design workshops for a suicide prevention app [25]. Moreover, following a literature review of research into mental health apps, Bakker et al [26] reported several recommendations for developing apps. This included customization options so the user can remain autonomous and the app is tailored to individual needs. There is considerable evidence base endorsing apps being customizable, and this research has further validated this.

One concern raised in the workshop was the possibility of being triggered by distressing content within peer support, including references to suicidality. This challenge was also noted by participants in O’Leary et al’s [11] study who used technology-based peer support for a range of mental health issues. The risks of peer support should be acknowledged alongside benefits and steps should be taken to mitigate these. As O’Leary et al [11] conclude, this can include training peers to help keep support spaces safe. Machine learning is also suggested as a possible solution to help signpost appropriate services or peers that may be able to offer support.

Following on from the above, training specialist peers to support those experiencing severe distress was discussed in this workshop as a way of protecting everyone involved. Participants felt that the training should mirror that of similar peer support services and that the specialist peers may be mental health professionals (such as counselors), psychology students, or even app users. Following this, it was also clear that users must be aware of who is supporting them. A systematic review of barriers to young people seeking mental health support identified that trusting the source of support is key [27], and it has been suggested that young people are more likely to seek support from their peers rather than mental health professionals [28]. One example of why this may be is that some young people report concerns that they may not be taken seriously by professionals [29]. Cumulatively, this underscores the need for young people to be certain of who they are speaking with and what they can expect from them, to ensure that they are comfortable engaging. Consequently, the importance of being clear and transparent with users was voiced in this workshop.

Participants in the workshop challenged the conceptualization of “progress” in the context of apps measuring whether an individual’s mental health is improving. This reframing of “progress” has been proposed elsewhere in the mental health literature. One example is within self-harm research, where understanding progress as a reduction or cessation of self-harm behaviors is becoming outdated in favor of more holistic approaches [30-32]. People with lived experience of self-harm have reported prioritizing other indicators of improvement, such as learning new coping skills or emotion regulation techniques. This resonates with the current findings, in which progress was a multifaceted concept encompassing learning skills, as well as improvements in lifestyle or emotions. The heterogeneity of young people at risk of suicide is exemplified here, and this should be acknowledged when delivering and evaluating digital peer support.

### Limitations

A key strength of this workshop was the opportunity provided for both group discussions and anonymous, individual reflections. Asking participants to anonymously evaluate the workshop was also a positive aspect as it indicated their comfort and confidence in sharing their perspectives, increasing the validity of these findings. A further strength was the involvement of young people with experience of both suicidal thoughts and of providing peer support.

The only mental health academics participating in the workshop also facilitated it and comprised of the research team. While we did contribute equally to conversations, we also facilitated the discussions which may have influenced the data collection process and limited our abilities to be fully immersed in the workshop. In addition, performing data analysis on our own contributions may have been subject to biases. However, Braun and Clarke [33] propose that the subjective experiences that researchers bring are a tool as opposed to a bias, so our involvement in the workshop meant that we were able to contribute to the discussions and also able to have first-hand experience to guide the analysis process. Group discussions and reflection were used to inform the analysis to improve the rigor. The 4 young people who participated in this workshop provided relevant and thorough insights indicative of a high level of information power [34]. Demographic information was not collected from participants, but across the workshop, there seemed to be heterogeneity with regard to demographics such as gender identity and age. To build on this, further data collection would be helpful from a more diverse group of participants to also understand the experiences of people representing different age groups, genders, and ethnicities. Last, the perspective of the 2 clinical staff was invaluable and it may also have been beneficial to have a wider variety of clinical stakeholder input, reflecting the breadth of clinical staff supporting young people in distress therapeutically.

It is worth noting that the presence of the Tellmi technical staff during discussions could have influenced how critical participants with lived experience were willing to be about the app features discussed. However, anonymous feedback from the workshop evaluation (Multimedia Appendix 2) indicated that people felt comfortable sharing their opinions, and this is reflected in the workshop findings in which concerns and challenges regarding app features were indeed raised.

### Conclusions

This study explored ways to develop and deliver suicide prevention tools in the context of digital peer support for suicide prevention among young people. The workshop also further corroborated the essential insight that young people with experience of suicidality have, as well as the value of multidisciplinary collaborations. To overcome some of the limitations in this study, seeking the perspectives of a greater variety of clinicians and external mental health academics may be beneficial in future research. Researchers should also consider implementing the suggestions put forth in this research when developing digital suicide prevention tools for young people. In particular, tools should be customizable, visually appealing, and transparent about the support that can and cannot be offered, and developers should work with users to develop content and language. This would help to validate these suggestions in practice.

## Appendix

Multimedia Appendix 1 Sample of candidate ideas.

Multimedia Appendix 2 Workshop evaluation.

Multimedia Appendix 3 Table of codes and themes.

## Data Availability

The data sets generated during this study are available from the corresponding author on reasonable request.

## References

[1] World Health Organization (2021). Suicide Worldwide in 2019: Global Health Estimates.

[2] (2021). Suicides in England. Samaritans.

[3] Bould H, Mars B, Moran P, Biddle L, Gunnell D (2019). Rising suicide rates among adolescents in England and Wales. Lancet.

[4] Hofstra E, van Nieuwenhuizen C, Bakker M, Özgül D, Elfeddali I, de Jong SJ, van der Feltz-Cornelis CM (2020). Effectiveness of suicide prevention interventions: a systematic review and meta-analysis. Gen Hosp Psychiatry.

[5] Zalsman G, Hawton K, Wasserman D, van Heeringen K, Arensman E, Sarchiapone M, Carli V, Höschl C, Barzilay R, Balazs J, Purebl G, Kahn JP, Sáiz PA, Lipsicas CB, Bobes J, Cozman D, Hegerl U, Zohar J (2016). Suicide prevention strategies revisited: 10-year systematic review. Lancet Psychiatry.

[6] McMenamy JM, Jordan JR, Mitchell AM (2008). What do suicide survivors tell us they need? results of a pilot study. Suicide Life Threat Behav.

[7] Rodway C, Tham SG, Ibrahim S, Turnbull P, Windfuhr K, Shaw J, Kapur N, Appleby L (2016). Suicide in children and young people in England: a consecutive case series. Lancet Psychiatry.

[8] Libon J, Alganion J, Hilario C (2023). Youth perspectives on barriers and opportunities for the development of a peer support model to promote mental health and prevent suicide. West J Nurs Res.

[9] (2019). Adults: media use and attitudes report 2019. Ofcom.

[10] Torok M, Han J, Baker S, Werner-Seidler A, Wong I, Larsen ME, Christensen H (2020). Suicide prevention using self-guided digital interventions: a systematic review and meta-analysis of randomised controlled trials. Lancet Digit Health.

[11] O'Leary K, Bhattacharya A, Munson SA, Wobbrock JO, Pratt W (2017). Design opportunities for mental health peer support technologies.

[12] Larsen ME, Nicholas J, Christensen H (2016). A systematic assessment of smartphone tools for suicide prevention. PLoS One.

[13] Tang PC, Smith MD, Adler-Milstein J, Delbanco T, Downs SJ, Mallya GG, Ness DL, Parker RM, Sands DZ (2016). The democratization of health care: a vital direction for health and health care. NAM Perspect.

[14] Lwembe S, Green SA, Chigwende J, Ojwang T, Dennis R (2017). Co-production as an approach to developing stakeholder partnerships to reduce mental health inequalities: an evaluation of a pilot service. Prim Health Care Res Dev.

[15] Hill C, Martin JL, Thomson S, Scott-Ram N, Penfold H, Creswell C (2017). Navigating the challenges of digital health innovation: considerations and solutions in developing online and smartphone-application-based interventions for mental health disorders. Br J Psychiatry.

[16] Marshall JM, Dunstan DA, Bartik W (2019). The digital psychiatrist: in search of evidence-based apps for anxiety and depression. Front Psychiatry.

[17] Mead S, Hilton D, Curtis L (2001). Peer support: a theoretical perspective. Psychiatr Rehabil J.

[18] (2023). Peer support in suicide prevention. Centre for Suicide Prevention.

[19] Ravaccia GG, Johnson SL, Morgan N, Lereya ST, Edbrooke-Childs J (2022). Experiences of using the digital support tool MeeToo: mixed methods study. JMIR Pediatr Parent.

[20] Alshurafa N, Lin AW, Zhu F, Ghaffari R, Hester J, Delp E, Rogers J, Spring B (2019). Counting bites with bits: expert workshop addressing calorie and macronutrient intake monitoring. J Med Internet Res.

[21] Van Velthoven MH, Cordon C (2019). Sustainable adoption of digital health innovations: perspectives from a stakeholder workshop. J Med Internet Res.

[22] Braun V, Clarke V (2006). Using thematic analysis in psychology. Qual Res Psychol.

[23] Braun V, Clarke V (2019). Reflecting on reflexive thematic analysis. Qual Res Sport Exerc Health.

[24] Jones RB, Stallard P, Agha SS, Rice S, Werner-Seidler A, Stasiak K, Kahn J, Simpson SA, Alvarez-Jimenez M, Rice F, Evans R, Merry S (2020). Practitioner review: co-design of digital mental health technologies with children and young people. J Child Psychol Psychiatry.

[25] Hetrick SE, Robinson J, Burge E, Blandon R, Mobilio B, Rice SM, Simmons MB, Alvarez-Jimenez M, Goodrich S, Davey CG (2018). Youth codesign of a mobile phone app to facilitate self-monitoring and management of mood symptoms in young people with major depression, suicidal ideation, and self-harm. JMIR Ment Health.

[26] Bakker D, Kazantzis N, Rickwood D, Rickard N (2016). Mental health smartphone apps: review and evidence-based recommendations for future developments. JMIR Ment Health.

[27] Gulliver A, Griffiths KM, Christensen H (2010). Perceived barriers and facilitators to mental health help-seeking in young people: a systematic review. BMC Psychiatry.

[28] Rickwood DJ, Deane FP, Wilson CJ (2007). When and how do young people seek professional help for mental health problems?. Med J Aust.

[29] Lavis A, Winter R (2020). #Online harms or benefits? an ethnographic analysis of the positives and negatives of peer-support around self-harm on social media. J Child Psychol Psychiatry.

[30] Owens C, Fox F, Redwood S, Davies R, Foote L, Salisbury N, Williams S, Biddle L, Thomas K (2020). Measuring outcomes in trials of interventions for people who self-harm: qualitative study of service users' views. BJPsych Open.

[31] Knowles S, Sharma V, Fortune S, Wadman R, Churchill R, Hetrick S (2022). Adapting a codesign process with young people to prioritize outcomes for a systematic review of interventions to prevent self-harm and suicide. Health Expect.

[32] Lewis SP, Hasking PA (2021). Self-injury recovery: a person-centered framework. J Clin Psychol.

[33] Braun V, Clarke V (2022). Conceptual and design thinking for thematic analysis. Qual Psychol.

[34] Malterud K, Siersma VD, Guassora AD (2016). Sample size in qualitative interview studies: guided by information power. Qual Health Res.

