# Supplementary material for: Developing Suicide Prevention Tools in the Context of Digital Peer Support: Qualitative Analysis of a Workshop With Multidisciplinary Stakeholders
Source: JMIR Form Res. 2023 Sep 20;7:e47178. doi: 10.2196/47178 (PMC10551794; doi:10.2196/47178)
Supplement: Multimedia Appendix 1 [file formative_v7i1e47178_app1.docx]

## Multimedia Appendix 1: Sample of Candidate Ideas

Below are 9 examples of the candidate ideas from both blocks discussed in the workshop, and a graph showing how the idea was rated on the two scales across the different stakeholder groups (technical staff from Tellmi, young people with lived experience, mental health practitioners, and mental health academics). Scale 1 evaluated the potential positive impact from 1 (no positive impact) to 4 (very positive impact). Scale 2 evaluated the extent to which the ideas needed to be discussed before implementation, from 1 (no discussion required) to 4 (needs much discussion).

**Real World Identification**

Collecting more information from app users (with consent) to help police locate app users at immediate and urgent risk.

**Positive Affirmations**

Notifications within the app designed to inspire optimism amongst app users by providing positive affirmations (eg, text or quotes).

**Direct Messages Between App Users and Counselors**

Allowing a private channel of communication between app users and trained counselors who can provide specialized support for high-risk users.

**High Risk User Stories**

This idea is to include personal stories in the directory from people who have struggled with suicidal thoughts or behaviors, and their experiences of managing these.

**Streamlined Sign Up Process**

Considering what aspects of the signup process could be condensed to make this shorter and easier for app users.

**Progress Tracking**

Built-in app features that help to measure mental health and any changes over time.

**Incentivising Desired Behavior**

User’s receiving rewards or awards for engaging in desired behaviors (eg, replying to posts) to help encourage engagement.

**Incentivising desired behaviour**

User’s receiving rewards/awards for engaging in desired behaviours (e.g., replying to posts) to help encourage engagement.

**Linking Accounts by Devices**

Being able to link different accounts made by the same user to help store their app history in one place.

**Refleciton Tools (Mood Journal)**

Giving users the option to reflect on and track their moods and experiences.
