# Supplementary material for: Developing Suicide Prevention Tools in the Context of Digital Peer Support: Qualitative Analysis of a Workshop With Multidisciplinary Stakeholders
Source: JMIR Form Res. 2023 Sep 20;7:e47178. doi: 10.2196/47178 (PMC10551794; doi:10.2196/47178)
Supplement: Multimedia Appendix 3 [file formative_v7i1e47178_app3.docx]

Multimedia Appendix 3: Table of example codes and themes.

| Theme | Example codes |
| --- | --- |
| The aims of the app must be clear and consistent | “transparency about what can be offered”  “aims need to be consistent”  “need to retain app ethos”  “needs to stay peer support” |
| There are unique considerations for supporting high-risk users | “possibility of triggering users”  “language can be stigmatizing”  “user needs to feel cared for”  “important to feel listened to” |
| “Progress” is a broad and multifaceted concept | “reflection is better than progress”  “progress can be demoralizing”  “progress is a broad domain”  “progress is more than mood” |
| Considering the role of those providing support | “need to keep boundaries”  “volunteers should be protected”  “peers need support”  “peer supporters need training” |
